# Evaluation of poly(N-isopropylacrylamide)/tetraphenylethylene/amphotericin B-based visualized antimicrobial nanofiber wound dressing for whole skin wound healing in rats

**DOI:** 10.1016/j.heliyon.2022.e12063

**Published:** 2022-12-06

**Authors:** Xinhui Zhai, Zongyao Cui, Yali Li, Shuang Hou, Weiyang Shen

**Affiliations:** a School of Science, China Pharmaceutical University, Nanjing 211198, China; b Ministry of Education Key Laboratory of Drug Quality Control and Pharmacovigilance, China Pharmaceutical University, Nanjing 211198, China; c Department of Pharmaceutics, China Pharmaceutical University, Nanjing 211198, China

**Keywords:** Hydrogel, Wound dressings, Local antibiotic delivery, Electrostatic spinning nanofiber membrane, Visualization

## Abstract

The aim of this work is to develop a novel nanofiber wound dressing with multiple functional properties that combines suitable mechanical properties, slow and controlled drug release, antifungal activity, and visual drug monitoring to accelerate wound healing while reducing systemic circulation of the drug, achieving reduced dose and side effects, and achieving patient satisfaction and compliance. In this paper, visualized nanofiber films were prepared using electrostatic spinning technology. This nanofiber wound dressing has soft tissue-like mechanical and antifungal properties and is biocompatible. In particular, the poly(N-isopropylacrylamide) (PNIPAAm)/tetraphenylethylene (TPE)/amphotericin B (AMB) nanofiber films showed good performance in terms of antifungal activity and cytocompatibility compared with medical gauze, and significantly accelerated the wound healing process in a mouse total wound defect model with PCL+PVP+TPE+AMB+PNIPAAm. The wound healing rate of nanofibrous membrane group was 100% at 14 days. In addition, histological analysis, collagen deposition and immunohistochemistry showed, for example, fewer inflammatory cells, more fibroblasts around the damaged area, increased wound epithelial atrophy, reduced granulation tissue, connective tissue reconstruction, epithelial tissue formation, and abundant small angiogenesis in the dermis near the epidermis; a higher level of collagen deposition fraction of 49.97%; and a simultaneous reduction in HIF-1α production and upregulated the expression of CD31. In conclusion, this antifungal nanofiber film showed promising applications throughout the skin wound healing process.

## 1 Introduction

Fungal drug resistance is one of the three major public health threats facing the world today. Its occurrence leads to longer hospital stays, increased health care costs, and higher mortality rates [1]. Exposure to chronic hard-to-heal wounds greatly increases the risk of infection for patients. Candida is one of the most common fungal pathogens of skin, mucous membrane and invasive fungal infections in humans and the fourth most common cause of hospital-acquired infections [2, 3]. Among them, Candida albicans is an opportunistic pathogen co-infecting the skin of most healthy hosts with the highest prevalence among a dozen Candida species that can cause disease. In immunocompromised patients, Candida albicans biofilms are resistant to antifungal drugs, and the disease caused by abnormal proliferation of Candida albicans is difficult to cure and prone to recurrence. Systemic infection is often life-threatening, with a mortality rate of 46%–75% [4, 5].

Amphotericin B has a significant inhibitory effect on Candida, with selective binding of ergocalciferol on the Candida cell membrane to increase membrane permeability. The AMB molecule consists of a hydrophobic region (polyolefin chain) and a hydrophilic region (polyhydroxy chain), with significant antifungal effect. About eight amphotericin B molecules form barrel-shaped pores bound by two end-to-end hydrogen bonds through hydrophobic interactions between their polyolefin chains and eight ergosterol molecules, each barrel-shaped pore consisting of eight polyene monomers that are arranged like a pentamer along the circumference of the barrel-shaped pore, with the hydrophilic polyhydroxyl chains facing the interior of the pore. The formation of such pores leads to a rapid outflow of K^+^, which inhibits fungal glycolysis and subsequent Mg^2+^ outflow. The ion outflow and subsequent proton inward flow lead to acidification of the fungal cell interior, cytoplasmic precipitation and eventual death [6, 7].

Temperature-sensitive polymers, which have excellent absorption and expansion capabilities and respond to external temperature stimuli, have been widely used in drug delivery systems. With the rapid development of nanotechnology, electrostatic spinning technology has received more and more attention from researchers in recent decades and has a good prospect in the biomedical field. As a new drug release dosage form, temperature-sensitive hydrogel electrostatically spun nanofiber membrane can intelligently achieve the release of loaded drugs by adjusting the external temperature to make the temperature-sensitive hydrogel respond to stimulation. In recent years, temperature-sensitive electrospun nanofiber membranes have been studied, for example, Yin et al [8] designed a polyurethane fiber membrane with a "sandwich" sandwich structure, which could achieve slow release of drugs for more than 144 h. Lv et al. [9] prepared a core-shell structured nanofiber with PNIPAAm temperature-sensitive hydrogel as the core layer and hydrophobic ethylcellulose (EC) as the shell layer using coaxial electrostatic spinning technique for the slow release of drugs, especially water-insoluble drugs. Li et al [10] prepared PNIPAAm/poly l-lactic acid-co-ϵ-caprolactone (PLCL) blended nanofibers loaded with the antibacterial drug ciprofloxacin (CIF). Due to the presence of PNIPAAm, the hydrophobicity of the fibers can be altered by adjusting the temperature, which in turn controls cell adhesion and separation, mitigates skin injury from dressing changes, and promotes wound healing. Wang et al [11] designed silver nanoparticle/poly N-isopropylacrylamide (AgNP/PNIPAAm) electrospun nanofiber membranes for detecting molecules, thereby enhancing the SERS signal, which Guo et al. [12] prepared a renewable nanomaterial for fluorometric immunoassay by adsorbing antibody-coupled ZnS quantum dots (ZQD-antiHA) onto PNIPAAm-grafted poly(2,6-dimethyl-1,4-phenylene propylene oxide) (PPO) electrospun fibers with high sensitivity and selectivity, which can be used for materials for bioanalysis, antigen detection, and fluorescent probes.

An antimicrobial dressing acts as a wound covering, replacing damaged skin and acting as a temporary barrier that can effectively inhibit the growth of bacteria or fungi or even kill them. It is the first barrier against bacteria or fungi. However, few dressings can simultaneously achieve the effect of not only controlling infection, but also encouraging cell proliferation and promoting wound healing. Therefore, research and development in related fields is essential to improve the health of all humans [13, 14, 15]. Electrospinning technology allows the production of fibers with a wide range of continuity in the nanometer range [16]. This technique can be used to manufacture composite electrospun nanofiber films using natural and synthetic polymers as a new method of topical drug delivery to address fungal infections. Nanofibers prepared by electrospinning have the advantages of large specific surface area, adjustable porosity, good ductility, and the ability to be loaded with drugs or other biomolecules [17]. Given these excellent properties of electrospinning technology, nanofibers are used for biomedical applications and regenerative drug therapy [18, 19], such as wound nanofiber tissue materials, slow and controlled drug release, and targeted drug delivery [20]. In recent years, many studies have also been conducted for wound healing dressings, bacterial fungal infections, and nanofibrous membranes, and the main research advances are shown in Table 1. PCL is a biodegradable polymer for tissue engineering [21, 22]. Due to the low degradability of PCL and the poor solubility of AMB, water-soluble PVP was mixed with PCL and successfully applied in this experiment [23].

**Table 1:** Research advances in wound healing, antibacterial, and nanofiber membrane related aspects.

Methods	Results	References
Preparation and characterization of nanosilver using curcumin-hydroxypropyl-β-cyclodextrin hydrate and its loading into bacterial cellulose hydrogels with wet wound healing properties.	The new dressing also has antibacterial activity against three common wound infection pathogenic microorganisms, Staphylococcus aureus, Pseudomonas aeruginosa and Candida.	[24]
Extraction of D. hansenii cultures from injured mice and inflammatory tissues of Crohn's fungal flora	A fungus parasitic in inflamed celiac disease tissue, D. hansenii, was identified to impair mucosal healing through the myeloid cell-specific type 1 interferon-CCL5 axis, which can lead to dysregulation of mucosal healing.	[25]
Electrospun nanofiber membranes based on chitosan with hull extract of mangosteen (GM) added to the membranes.	Compared to the control group (gauze covered), the nanofiber film had an antibacterial effect and accelerated the healing rate in the wound healing test, and the α-mannoside content was maintained at 90% after 3 months.	[26]
Ultra-transparent porous cellulose membranes (CMs) of chitosan-coated nanofibers prepared using a simple one-step electrostatic spinning technique	CM-CS has good antibacterial activity against Escherichia coli and Staphylococcus aureus, and has a good effect on promoting wound healing.	[27]
Combining the good mechanical properties of poly(ε-caprolactone) (PCL) and the multiple functions of QCSP, a series of antibacterial, antioxidant and electroactive nanofiber membranes were prepared by electrostatic spinning of quaternized polymer solutions of poly(ε-caprolactone) (PCL) and chitosan-grafted polyaniline (QCSP).	PCL/QCSP10, PCL/QCSP15 and PCL/QCSP20 showed 70%, 86% and 92% killing of S. aureus and 82%, 94% and 95% killing of E. coli, respectively. wounds treated with PCL/QCSP15 NFM showed less inflammatory infiltration, higher density of fibroblasts, thicker granulation tissue and high collagen deposition, and rapid wound healing.	[28]
Chitosan-polyethylene oxide (PEO) nanofibers for topical antibiotic delivery of teicoplanin	Drug release up to 12 days, 1.5 ∼ 2-fold improved killing of Staphylococcus aureus, and significant improvement in wound healing with nanofibers containing 4% teicoplanin.	[29]
A facile method for the one-pot synthesis of antimicrobial hydrogel trauma dressings from polyvinylpyrrolidone acrylamide 1-vinyl-3-butylimidazole and polyethylene glycol dimethacrylate	AH-1, AH-2 and AH-3 showed significant antibacterial activity against E. coli, S. aureus and Candida albicans, respectively, with the colony count tending to zero as the proportion of VBIMBr fragments increased (from AH-1 to AH-3). Notably, 100% of the bacteria were killed after 24 h of exposure to AH-3. In addition, AH-1 and AH-2 inhibited E. coli, S. aureus and Candida albicans by more than 98%.	[30]
Ag/ZnO loaded chitosan composite dressing	It has significant antibacterial activity against drug-sensitive Escherichia coli, Staphylococcus aureus and Pseudomonas aeruginosa. The chitosan-Ag/ZnO dressing exhibited faster wound healing, more complete re-epithelialization and denser collagen deposition properties.	[31]

For Candida albicans infection, there are still many problems in the treatment of oral antifungal drugs ketoconazole and clotrimycin, such as the drugs do not directly act on the skin tissue, there is no targeted drug delivery, and the bioavailability is low; Drug resistance and poor treatment effect; No slow and controlled release function; And the drug release effect needs to be monitored by analyzing blood or urine, which is not convenient for operation. Therefore, new wound dressings need to be designed to solve these problems. These extraordinary regulators can be directly inserted into the focus of infection, providing controlled and long-term release of antimicrobials after a single dose, reducing the frequency of administration. By reducing the number of drugs taken and avoiding the systemic circulation of drugs, the possibility of any side effects and drug interactions can be reduced/eliminated, which can greatly improve the satisfaction and compliance of patients, and at the same time, rapid detection of drug release can be achieved.

In this context, amphotericin, TPE, and PNIPAAm were used for the first time to synthesize electrospun nanofiber membranes with internal filtration effect, controllable drug release temperature and pH changes, and visualized drug release effects. As a polyene, Amphotericin B is very effective against candida albicans and non-candida albicans. It binds to ergosterol on the fungal cell membrane to form a transmembrane pore. Thus, the membrane is depolarized and its permeability to univalent protons and cations increases with the flux of intracellular molecules to the external environment. At the same time, the effect of pH value and temperature change on the total release of PNIPAAm was studied. The nanofiber membrane can reduce the dose and side effects of the drug, improve the therapeutic effect, and monitor the release of the drug in real time to achieve visual control of drug release. In addition, fungal, cytocompatibility and animal experiments showed that 37 °C and pH = 6.5 had the best antibacterial effect, which could promote wound healing to a certain extent.

## 2 Materials and methods

### 2.1 Materials

N-isopropylacrylamide (NIPAAm) and N, N′-methylenebis(acrylamide) (BIS) were obtained from Beijing Bailingwei Technology Co., Ltd. (Beijing, China). N, N, N′, N′-tetraethylethylenediamine (TEMED) was purchased from Alfa Aesar (Shanghai, China). Potassium persulfate (KPS) and polycaprolactone (PCL, Mn 80, 000 Da) were purchased from Sigma-Aldrich. Polyvinylpyrrolidone (PVP, Mn 58, 000 Da) and 1,1′,1″,1‴-(1,2-Ethenediylidene)tetrakisbenzene (TPE) were purchased from McLin (Shanghai, China). Amphotericin B (AMB, 100.0%) and Dimethyl sulfoxide (DMSO) were purchased from Sigma-Aldrich. Acetonitrile (HPLC grade) was supplied by ROE SCIENTIFIC INC (Delaware, America). Phosphoric acid and Agar powder were supplied by McLin (Shanghai, China). Yeast extract, peptone, and glucose were provided by Oxoid (UK). PBS buffer was purchased from Kaiji Biological Co., Ltd. (Nanjing, China). NIH/3T3 cells were purchased from Shanghai Institute of Cells, Chinese Academy of Sciences. Fetal bovine serum, double antibody, DMEM medium and trypsin were purchased from GIBCO (USA). MTT reagent was purchased from FLUKA company (USA). Deionized water from the Ulupure Water Analyzer with a resistance of 18.5 MΩ was used throughout the study. N-hexane was purchased from Shanghai Titan Technology Co. Acetone was purchased from Nanjing Chemical Reagent Co.. Poly-N-isopropylacrylamide (PNIPAAm), Mν = 2.26 × 10^4^, was manufactured. Anhydrous ethanol, xylene, N-butanol, neutral gum, 3% hydrogen peroxide were purchased from Sinopharm Chemical Reagent Co.. N,N-dimethylformamide (DMF), tetrahydrofuran (THF) were purchased from Shanghai Lingfeng Chemical Reagent Co.. Candida albicans ATCC10231 was purchased from Solaibao Company. Physiological saline was purchased from Ningbo Mingzhou Biotechnology Co.. Rat blood was obtained from cultured rats. Citric acid (PH 6.0) antigen repair solution, 4% paraformaldehyde, bovine serum albumin (BSA), hematoxylin staining solution, hematoxylin differentiation solution, hematoxylin return blue solution, primary antibody: Hif-1α, primary antibody: CD31, secondary antibody: HRP goat anti-rabbit secondary antibody, and histochemistry kit DAB color developer were purchased from Servicebio. All other reagents were of analytical grade.

### 2.2 Fabrication of nanofibrous membranes

The electrospinning solution was prepared by dissolving 10% PCL, 10% PVP, 50 mg PNIPAAm (Please refer to the previous literature of this research group [32] for the preparation and characterization methods of this temperature-sensitive polymer.), 20 mg TPE and gradient content AMB in 5 mL DMF: CHCl_3_ (2:3, v/v) under vigorous stirring at room temperature. The homogeneous solution was filled into a 5 mL of plastic syringe fitted with a stainless needle (22 G). The syringe was fixed on the syringe pump (LSP-01-1A refraction pump, Baoding Lange Constant Flow Pump Co., Ltd.). The flow rate of electrospun solution was 0.5 mL/h and the applied voltage was 12.5 kV. The working distance between the needle tip and the collector was set at 12.5 cm. Nanofibers were collected on a roller coated with aluminium foil and dried under vacuum (50 °C, - 0.98 kPa) for 24 h to remove residual solvents.

### 2.3 Morphology and chemical properties of nanofibers

In this study, the prepared nanofiber membranes were characterized by the following methods. For all the features, at least three representative samples are available, and for more than three samples, the features are again labeled in the corresponding section.

#### 2.3.1 Fluorescence spectrophotometer

The masses of the prepared TPE and AMB were 10 mg. 1 mg, 10 mg. 2 mg, 10 mg: 3 mg, 10 mg: 4 mg, 10 mg: 5 mg, 10 mg: 6 mg, 10 mg: 7 mg, 10 mg 8 mg, 10 mg 9 mg, 10 mg. The prepared different nanofiber membranes were cut to the appropriate size and infiltrated in a deionized water soaked glass The excitation wavelength was set to 330 nm, slit 5, wavelength range 300–600 nm, and measured by fluorescence spectrophotometer.

#### 2.3.2 Fiber diameter measurement

The surface morphology of the fiber scaffolds was analyzed using SEM (Hitachi S-3400N, Japan). Square samples (0.5 cm × 0.5 cm) of various electrospun nanofiber films prepared in a sputter coating device (Hitachi E-1010) were cut and coated with gold, and then digital micrographs were taken from randomly selected sample areas at an accelerating voltage of 15 kV at a magnification of 5000x. The images were analyzed using NIH Image-J software to determine the diameter distribution of fibers in electrospun nanofiber films.

#### 2.3.3 Contact angle

The water contact angle (CA) of the samples was determined using a VCA Optima surface analysis system (AST Products, Inc., Billerica, MA, USA). Each membrane sample was cut into thin strips and the contact angle was measured by attaching both edges of the membrane to a glass slide using transparent tape. A minimum of five membrane strips were used for each contact angle measurement [33].

#### 2.3.4 Water absorption

The absorbency of the membranes was determined by weight method [34, 35]. First, the membranes were cut into small pieces of 2 cm × 2 cm, dried in a vacuum oven at 40 °C for 4 h, and their dry weights (Wd) were accurately measured. Soak in a glass bottle filled with PBS solution at pH 7.4 at 37 °C for 24 h. Remove the PBS and wipe the surface of the nanofiber pad with filter paper to remove excess water and weigh (Ww). These tests were performed at least three times (n ≥ 3). The expansion degree is calculated according to the following formula (1):

(1) Wateruptake（%）=[(Ww−Wd)/Wd]×100%(n=3)

#### 2.3.5 Fourier transform infrared spectrometer

Functional organic groups of three different electrospun films were analyzed and characterized using attenuated total reflection-Fourier transform infrared spectroscopy (ATR-FTIR, FT/IR-4600, JASCO, Japan). The nanofiber samples were mounted on a diamond window and the spectra were recorded in the spectral range of 4000 and 400 cm^−1^ with an average of 16 scans.

#### 2.3.6 Water vapor transmission rate

The water vapor transmission rate (WVT) of the membranes was determined according to the ASTM E96 standard method. Briefly, each membrane specimen was cut to the appropriate size you then sealed in the mouth of a cylindrical cup (2.75 cm diameter) containing 30 mL of deionized water. The films were tightly secured with barrier tape to prevent any water loss and weighed for their initial weight (Wi). The vial coated with the dressing was then placed in a constant temperature and humidity incubator (temperature 37 °C, relative humidity 79%) and the vial containing only 10 mL of deionized water was taken as a blank control. 48 h later, the vial was removed and weighed (Wf) and the measurements were repeated 5 times for each sample, and the water vapor transmission rate was calculated according to the following Eq. (2) [36].

The WVTR is calculated by the following formula:

(2) WVTR(gr/m2·day)=[(Wi−Wf)/A]×100%(n=3)

#### 2.3.7 Mechanical properties

The tensile strength of the prepared nanofiber films was tested at room temperature using a universal materials testing machine (Instron, USA). Rectangular strips of 0.15 mm thickness of each sample were used for analysis and the average of three samples was taken according to the American Standard for Testing Methods (ASTM) D882-10, the standard test method for tensile properties of nanofiber films. The crosshead speed and gauze length were 5 mm/min and 2 cm, respectively, using a 10 KN load. The measured values of tensile strength, Young's modulus, and elongation at break (%) of each nanofiber film sample were subsequently compared to the values reported for human skin.

### 2.4 In vitro studies

#### 2.4.1 Release profile assay

10 mg drug-loaded nanofibers were immersed into 10 mL of PBS (pH 6.5, 7.4). The vials were shaken at 100 rpm and incubated at 37 °C and 25 °C, respectively. At regular intervals, 250 μL of the release solutions were taken from the incubation medium and 250 μL fresh PBS at the same temperatures were added back to the dissolution medium. The released drug, AMB, was analysed by LC-2010 or CHT Shimadzu High Performance Liquid Chromatographer (Shimadzu, Japan) (HPLC) and the cumulative drug release degree was obtained by the standard curve (r > 0.999) (n ≥ 3). Chromatographic conditions of the drug were as follows:

Column (Agilent ZORBAX SB-Aq, 150 × 4.6 mm, 3.5 μm); mobile phase: Acetonitrile - Phosphoric acid solution (pH 1.00 ± 0.05) (37:63, v/v); detection wavelength: 383 nm; column temperature: 25 °C; flow rate: 0.8 mL/min; injection volume: 10 μL.

#### 2.4.2 Bacterial culture and antibiotic susceptibility testing

The experimental fungus was Candida albicans ATCC10231, and the agar flat diffusion method was used to evaluate the inhibitory performance of the nanofiber membrane against the fungus. The principle of this experiment was to spread the fungal solution with a concentration of about 1 × 10^8^ CFU/ml, evenly on YPD agar medium plates (pH 6.5 7.4, respectively), and then affix a circular sample with a diameter of 10 mm to the surface of the medium, and use a filter paper sheet of the same size as a blank control group, and incubate it at 37 °C, 25 °C in a water-tight incubator for 24 h to observe the inhibition circle.

#### 2.4.3 In vitro cytotoxicity test

The cytotoxicity of the nanofiber membrane was evaluated by NIH/3T3 fibroblasts in mice, and its biocompatibility was investigated. Firstly, nanofiber was sterilized under UV light, and then membrane extract was prepared (The membrane thickness is less than 0.5 mm, and the extraction ratio is 6 cm^2^/mL). In addition, NIH/3T3 was inoculated in 96 orifice plate and cultured in air of 37 °C and 5% CO_2_ for 24 h. The cell metabolism activity was measured by 3-(4,5-dimethylthiazole-2-yl)-2,5-diphenyltetrazolium bromide (MTT), and its absorbance was measured by bio-Tech (model elx808) at 492 nm. The strength of absorbance reflects the number of living cells. Repeat the whole process five times in parallel.

#### 2.4.4 Blood compatibility

The whole blood was anticoagulated and diluted with saline. 200 μL blood samples were poured into the membrane, cultured at 37 °C for 60 min, and centrifuged at 1500 rpm for 10 min. The absorbance value of each sample was read at 545 nm. The blood diluted with deionized water is considered to be a positive control, while the blood diluted with saline is considered to be a negative control.

Based on the measured absorbance value, the hemolysis rate of the test sample is calculated according to the following formula (3).

(3) $$HP\;\left(\%\right)=\left(D_{t}-D_{nc}/D_{pc}-D_{nc}\right)\times 100\%\;\left(n=3\right)$$

Wherein:

HP—the hemolysis rate of the sample.

D_t_—the absorbance value of the test sample.

D_nc_—the absorbance value of the negative control sample.

D_pc_—the absorbance value of the positive control sample.

### 2.5 In vivo assays

#### 2.5.1 Animals

This study was approved by the ethics committee of China Pharmaceutical University. In the present study, male SD rats (180–200 g) were used, and each animal was placed in a separate cage.

#### 2.5.2 In vivo wound model

After anesthesia, the back and neck of each rat were shaved, and residual hair was removed with depilatory cream and then wiped with 70% ethanol. Three wounds were created in the neck region of each rat using a skin biopsy punch (wound area of 1 cm^2^) (n = 3). Throughout the study period, the same size of drug-containing temperature-sensitive nanofiber membrane, blank nanofiber membrane, gauze, and drug-containing nanofiber membrane were placed on the wounds without removing the test material (the dressing change time for the drug-containing temperature-sensitive nanofiber membrane group was based on the change in membrane fluorescence intensity, while the other groups changed dressings every one or two days). The wound area was measured until the wound was completely healed. On days 3, 7, 10 and 14 after surgery, rats were randomly selected and sacrificed for further analysis.

#### 2.5.3 Wound healing efficiency of synthesized nanofibers

The changes in the wound area were evaluated by taking the digital images on days 3, 7, 10, and 14 and the images were processed with Image J 1.41.

Wound healing rate (%) = S0−Sd/S0×100.

Wherein:

S_0_—the initial area of postoperative wound.

S_d_—the area of wound on the day of dressing change.

#### 2.5.4 Histological evaluation

1. Paraffin sections dewaxed to water: sequentially put the sections into xylene Ⅰ for 15 min -xylene Ⅱ for 15 min - xylene III for 15 min - anhydrous ethanol Ⅰ for 5 min - anhydrous ethanol Ⅱ for 5 min - 85% alcohol for 5 min - 75% alcohol for 5 min - distilled water wash.
2. Antigen repair: Place the tissue section in a repair box full of citric acid antigen repair buffer (pH6.0) in a microwave oven for antigen repair, medium heat for 5 min, cease fire for 5 min and then low heat for 10 min. Excessive evaporation of the buffer should be prevent in this process. Do not dry the film. After natural cooling, the slides were placed in PBS (pH7.4) and washed 3 times by shaking on a decolorization shaker for 5 min each time.
3. Block endogenous peroxidase: Place the slides in 3% hydrogen peroxide solution, incubate them for 25 min at room temperature and avoid light. Place the slides in PBS (pH 7.4) and shake and wash them 3 times on a decolorized shaker for 5 min each time.
4. Serum closure: Add 3% BSA dropwise in the histochemical circle to cover the tissue evenly and close it at room temperature for 30 min (The primary antibody is closed with rabbit serum for goat source, and BSA for other sources).
5. Add primary antibody: Gently shake off the closure solution, add PBS on the section drop by drop in a certain proportion of the prepared primary antibody, and incubate the section flat in the wet box at 4 °C overnight (Add a small amount of water in the wet box to prevent antibody evaporation).
6. Add secondary antibody: Place the slides in PBS (pH 7.4) and shake and wash them 3 times on a decolorization shaker for 5 min each time. slightly shake the section dry and add the secondary antibody (HRP-labeled) of the corresponding species in the circle to cover the tissue and incubate them for 50 min at room temperature.
7. DAB color development: The slides were placed in PBS (pH7.4) and washed 3 times on a decolorization shaker for 5 min each time. The section was shaken dry and freshly prepared DAB color development solution was added dropwise in the circle. The color development time was controlled under the microscope; the positive color was brownish yellow; and color development was terminated by rinsing the section with tap water.
8. Re-staining cell nuclei: Hematoxylin re-staining was performed for about 3min, washed with tap water, differentiated with hematoxylin differentiation solution for a few seconds, rinsed with tap water, hematoxylin return blue solution returning blue, rinsing with running water.
9. Dehydration and sealing: Put the section into 75% alcohol for 5 min - 85% alcohol for 5 min - anhydrous ethanol Ⅰ for 5 min - anhydrous ethanol Ⅱ for 5 min - n-butanol for 5 min - xylene Ⅰ for 5 min to dehydrate transparently, take the section out of xylene to dry slightly, and seal the section with neutral gum.
10. Microscopic examination, image acquisition and analysis.

The main indicators include inflammatory cell infiltration, fibroblast migration, connective tissue synthesis and re-epithelialization [37].

#### 2.5.5 Collagen deposition analysis

Hydroxyproline content was estimated using a commercial kit to assess the amount of collagen in the wound.

#### 2.5.6 Statistical analysis

All results were statistically analyzed and expressed as mean ± standard deviation (SD). One-way ANOVA was used to determine statistical differences, followed by Bonferroni post hoc test in SPSS version 24 (IBM) for multiple comparisons. Differences were considered significant if P < 0.05 [38, 39].

## 3 Results and discussion

### 3.1 Characterization of nanofiber membranes

#### 3.1.1 Fluorescence spectrophotometer

**Figure 1:**
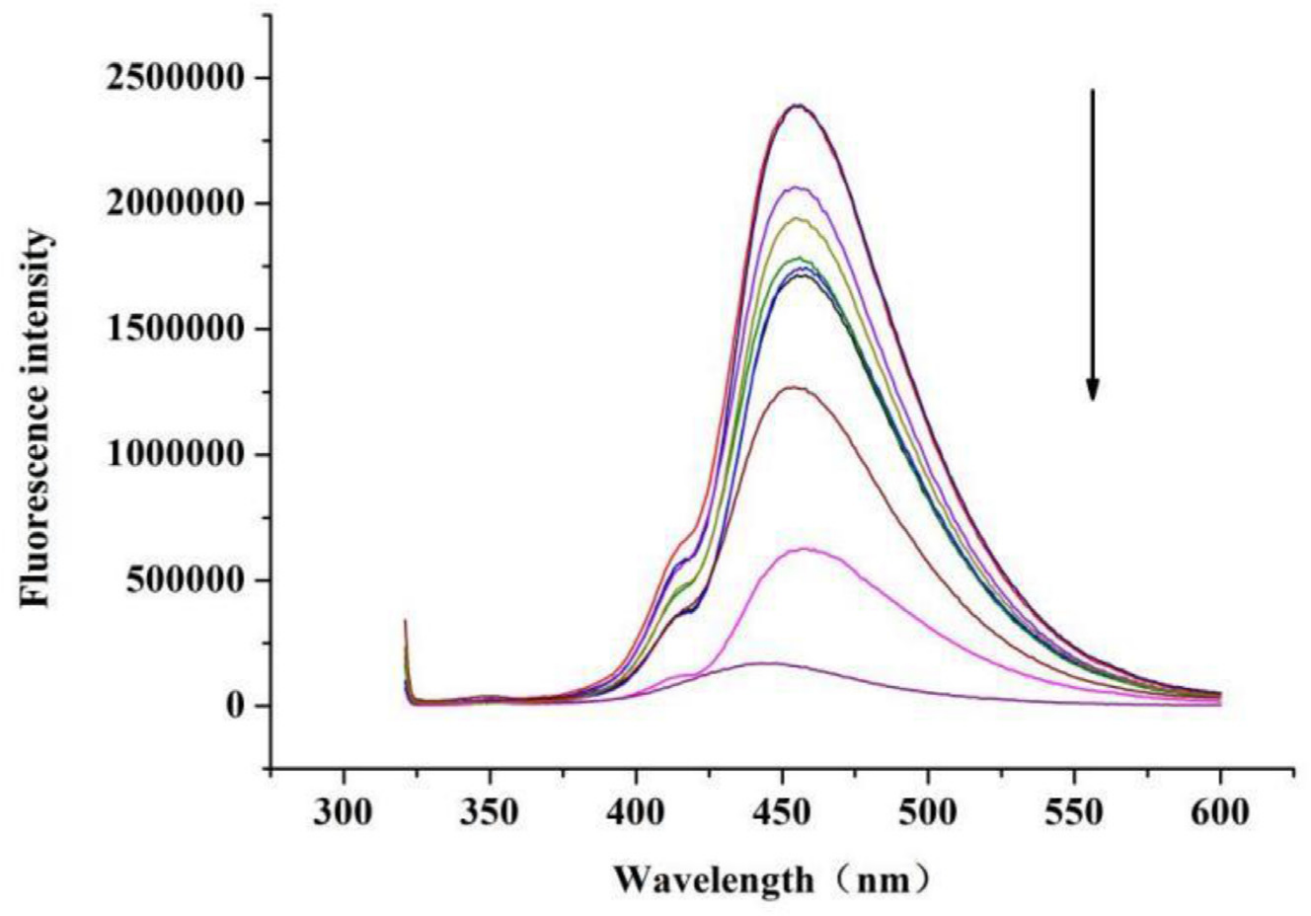
Nanofiber membrane TPE and AMB FL curve.

As seen in Figure 1, the fluorescence intensity of the nanofiber membrane gradually decreased with the addition of AMB to the spinning solution. the fluorescence intensity of TPE was stronger in the nanofiber membrane than in the solution. The reason for these two phenomena is due to the internal filtering effect of AMB and TPE within the nanofiber membrane. The UV absorption broad spectrum of AMB is closer to the excitation light wave of TPE, so with the increase of AMB content, the excitation light spectrum of TPE is absorbed away, resulting in a gradual decrease of the emitted fluorescence, and when AMB is released, the emitted light of TPE gradually increases again, which can be caused by the internal filtering effect fluorescence changes to reflect the degree of drug release [40]. TPE is a typical AIE fluorophore that does not emit light when dissolved in benign solvents, but in solids due to its restricted intramolecular motion leads to enhanced fluorescence intensity.

#### 3.1.2 Fiber diameter measurement

**Figure 2:**
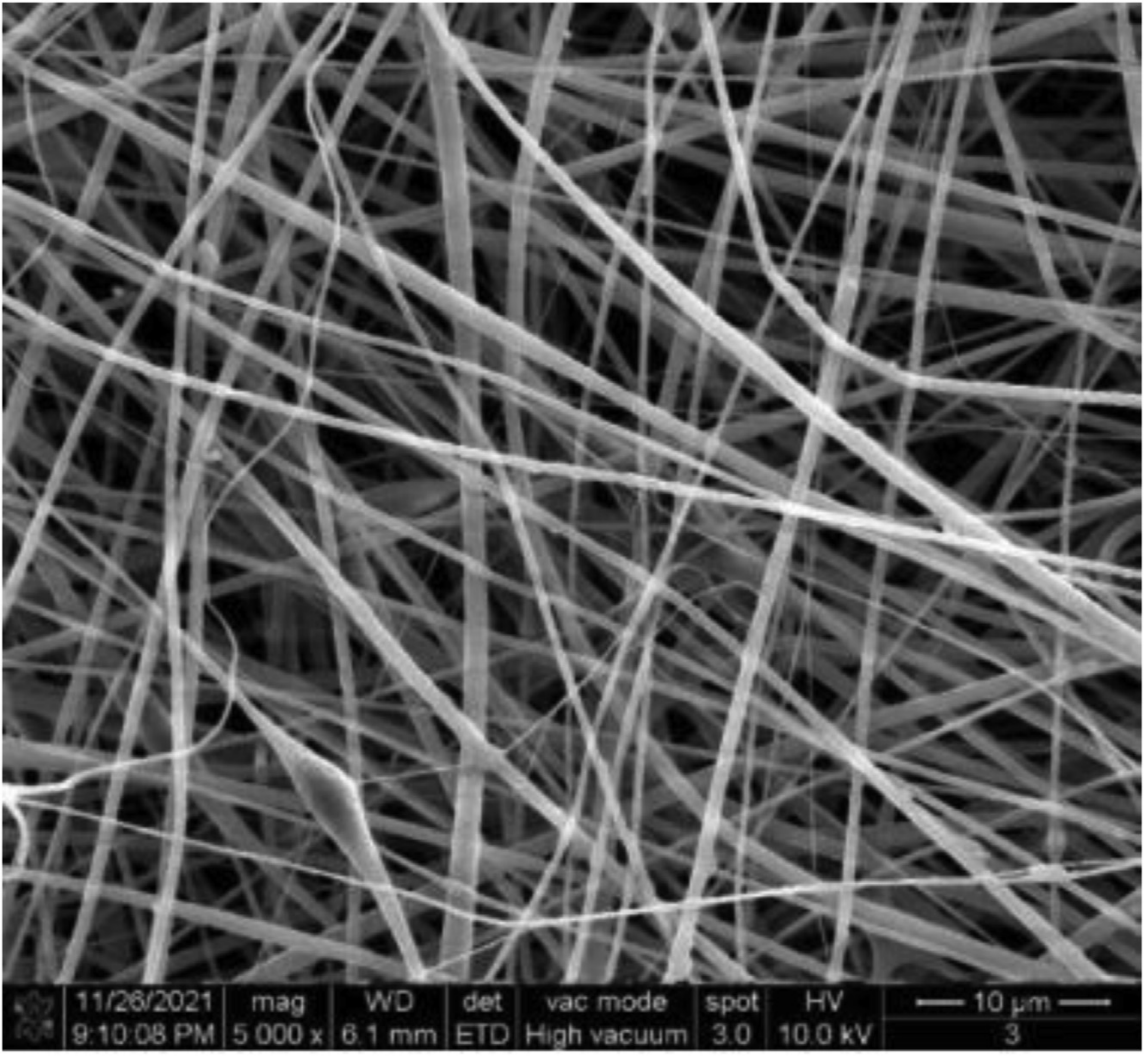
SEM image of PVP+PCL+AMB+TPE+PNIPAAm nanofiber membrane.

From Figure 2, it can be seen that the prepared temperature-sensitive polymer electrostatically spun nanofibers have smooth surface, uniform diameter and no bead structure, which indicates that the temperature-sensitive polymer electrostatically spun nanofibers have been successfully prepared.

#### 3.1.3 Contact angle

**Figure 3:**
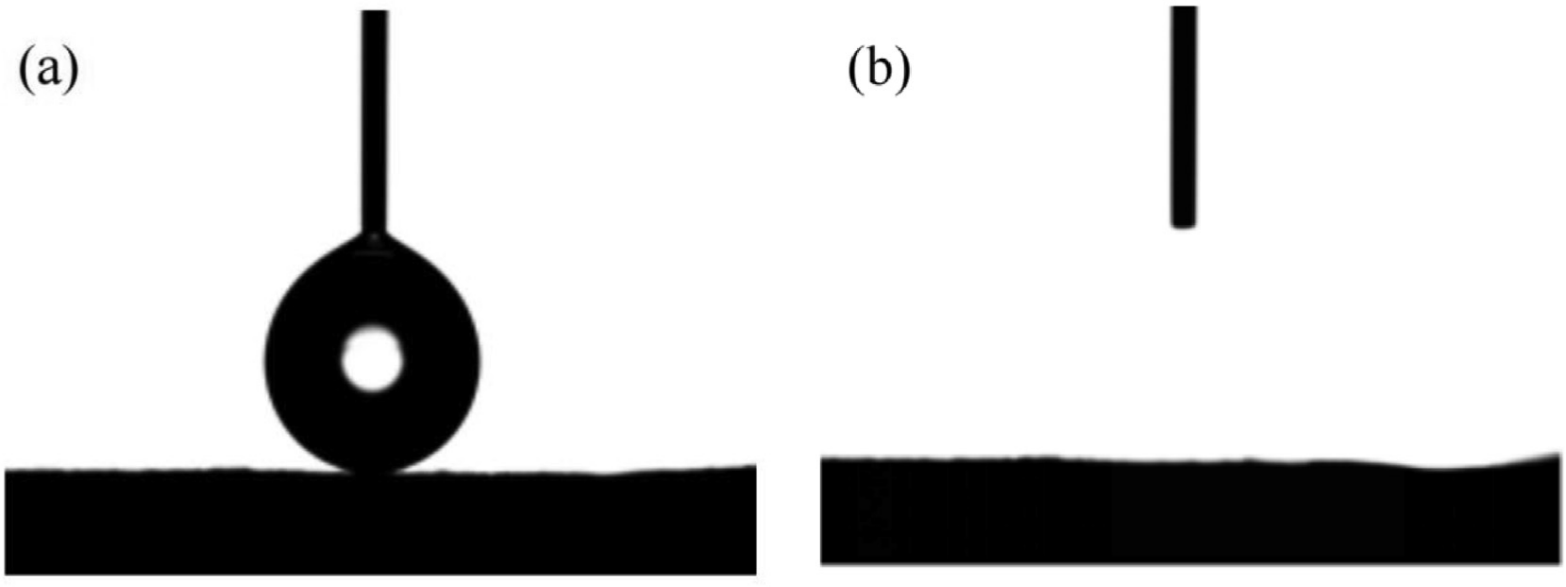
PVP+PCL+AMB+TPE+PNIPAAm nanofiber membrane WCTA plot (a) 0s. (b) 78s.

For hydrophilic materials, the contact angle decreases with the increase of absorbance [41, 42]. It can be seen from Figure 3 that the contact angle of the nanofiber membrane changed to 0° within 78 s, which indicates that the temperature-sensitive polymer nanofiber membrane has good hydrophilicity. The reason for this result may be that amphotericin B is poorly hydrophilic, while PCL is a hydrophobic polymer and PVP is a hydrophilic polymer, meanwhile, the temperature-sensitive polymer is hydrophilic water, but the hydrophilicity needs to be converted to produce in a certain time [43, 44].

#### 3.1.4 Fourier transform infrared spectrometer

**Figure 4:**
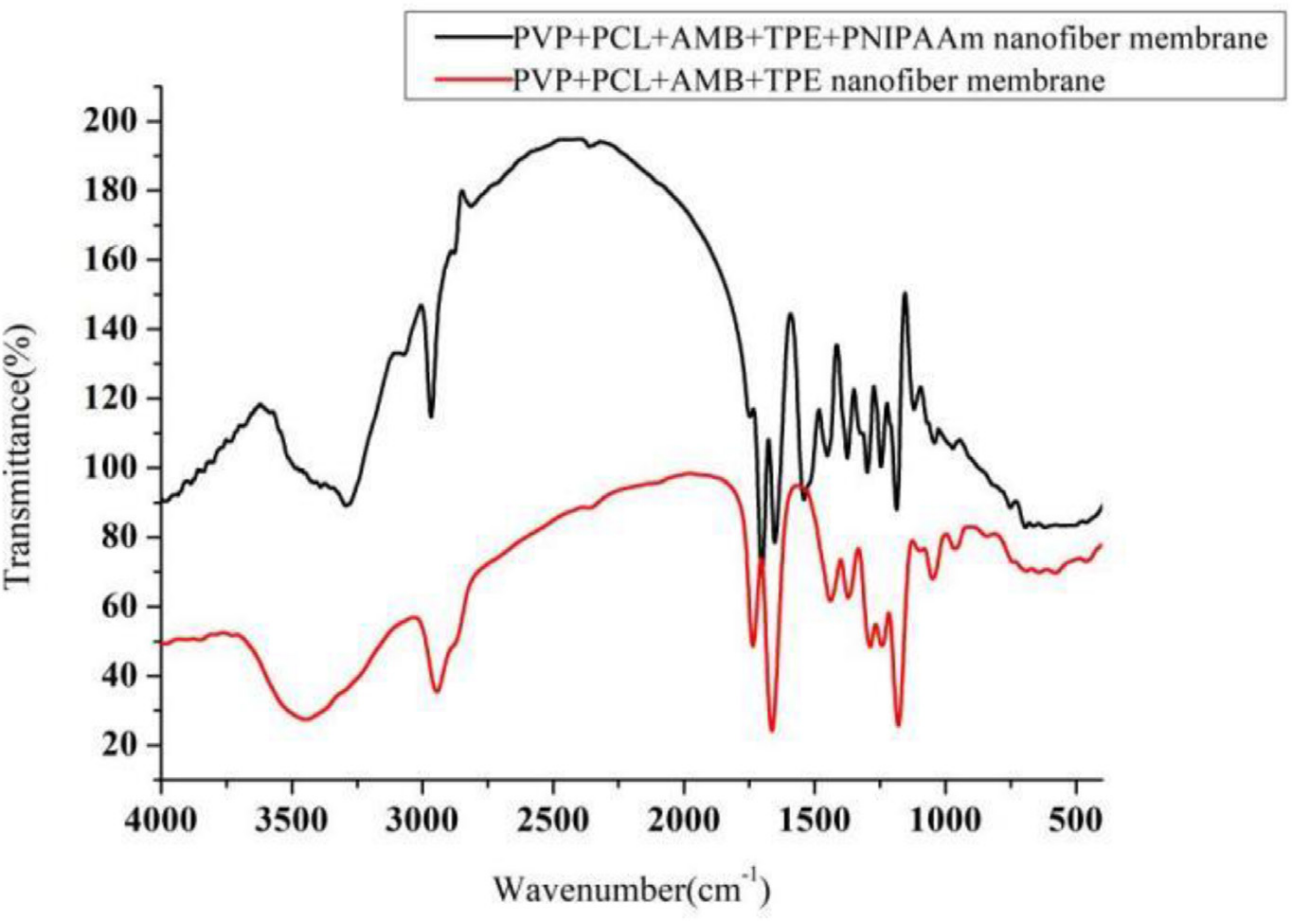
FT-IR map of nanofiber membrane.

As shown in Figure 4, the CH_2_ bending vibration of 1462 cm^−1^ amphotericin B, 1750 cm^−1^ bending vibration peak of ester and 1680 cm^−1^ twisted vibration peak of acid as well as 1200 and 1250 cm^−1^C-O-C stretching peaks of ester disappeared, indicating that PNIPAAm successfully wrapped the drug inside the gel.

#### 3.1.5 Water absorption and water vapor transmission rate

A moist environment promotes penetration of active substances, protects the wound from bacterial invasion, and provides painless debridement of the wound surface after recovery [45]. The ideal wound dressing should lock in exudate and maintain proper wound moisture during the healing process. The results of the data are shown in Tables 2 and 3. From Table 2, the absorption rates of the prepared nanofiber membranes can be calculated as 118%, 138% and 561%, respectively. From Table 2, the water vapor transmission rate of the blank group was 10136 ml/m^2^/day and the water vapor transmission rates of the nanofiber membranes were classified as 2773 ml/m^2^/day, 2461 ml/m^2^/day and 2855 ml/m^2^/day. According to the literature, the most suitable water vapor transmission rate values for nanofiber membranes are 2000–2500 ml/m^2^/day [46], and the prepared temperature-sensitive polymeric nanofiber membranes were exactly in this interval, indicating that the prepared temperature-sensitive. The water vapor transmission rate of the prepared temperature-sensitive polymer nanofiber membranes was good. The difference in water absorption capacity among the prepared nanofiber membranes can be explained by their respective porous structures. Water molecules are believed to be physically trapped by the fine network structure of the fibrous membranes [47]. The incorporation of temperature-sensitive hydrogels results in a denser surface and smaller porosity, which reduces water absorption. Complete evaporation of the adsorbed water takes approximately 48 h. In addition to water retention, water loss in open wounds is related to the water allowance of the dressing. According to Table 3, the water vapor allowable rates of AMB+PVP+PCL+TPE and PVP+PCL membranes were almost constant with no statically significant differences between them. The AMB+PVP+PCL+TPE+PNIPAAm nanofiber membrane group could maintain a suitable water environment for exudative wounds without excessive dehydration, probably due to the fact that PNIPAAm through strong hydrogen bonding interactions help to retain water molecules effectively.

**Table 2:** Weight comparison of nanofiber membrane before and after water absorption.

	W_d_ average value (g)	W_w_ average value (g)	Water absorption (%)
AMB+PVP+PCL+TPE nanofiber membrane	0.0126	0.0300	138
PNIPAAm+PVP+PCL+AMB+TPE nanofiber membrane	0.0056	0.0122	118
PVP+PCL nanofiber membrane	0.0079	0.0522	561

**Table 3:** Experimental data of water vapor transmission rate of nanofiber membrane.

	W_i_ average value (g)	W_f_ average value (g)	Water vapor transmission rate (ml/m^2^/day)
Blank group	24.9675	18.5986	10136
AMB+PVP+PCL+TPE nanofiber membrane	24.8402	23.0976	2773
PNIPAAm+PVP+PCL+TPE+AMB nanofiber membrane	25.4676	23.9214	2461
PVP+PCL nanofiber membrane	25.1375	23.3438	2855

#### 3.1.6 Mechanical properties

The wound dressing should maintain its integrity during use except in humid environments. The mechanical properties of the membranes are shown in Table 4. The good mechanical properties of the AMB +PVP+PCL+TPE+PNIPAAm nanofiber membranes are attributed to the addition of temperature-sensitive polymers, which are sufficiently tough to allow the dressing to adhere well to the wound site after incorporation [10].

**Table 4:** Mechanical strength of nanofiber membrane.

	PNIPAAm+AMB+TPE+PVP+PCL nanofiber membrane	AMB+TPE+PVP+PCL nanofiber membrane	PVP+PCL+TPE nanofiber membrane
Maximum force value（N）	3.28	3.36	1.66
Tensile strength（MPa）	0.16	0.16	0.08
Elongation(%)	51.72	22.96	19.02

### 3.2 In vitro studies

#### 3.2.1 Release profile assay

**Figure 5:**
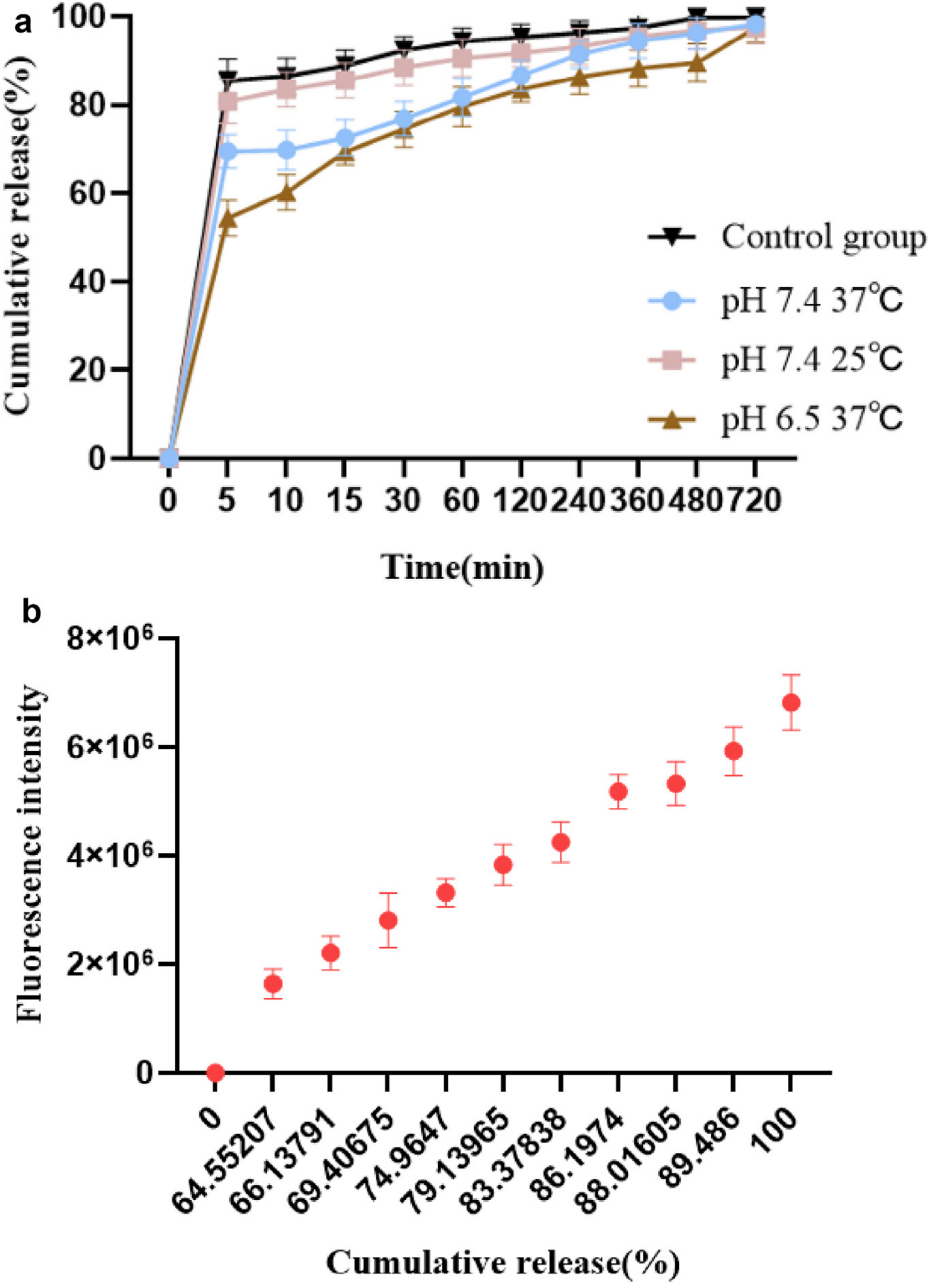
(a) Plot of cumulative drug release. (b) Plot of AMB drug release versus fluorescence intensity.

From Figure 5 (a), it can be seen that the drug release rate at 37 °C is slower than that at 25 °C. The delayed release time of the drug at 37 °C can reach 12 h, and the complete release of the drug at 25 °C is about 8 h, while the drug release time as a control group (nanofiber membrane without PNIPAAm) is 6 h. This indicates that the temperature-sensitive polymer has a good delayed release function. Due to the temperature-sensitive property of PNIPAAm, the PNIPAAm molecular chains curled at temperatures higher than LCST, and the contracted PNIPAAm polymer wrapped the drug more tightly, making it difficult for the drug to escape, thus delaying the drug release. It can also be seen from the figure that the drug release rate is different at different pH conditions, probably due to the positive charge of N atoms on the structure of the PNIPAAm temperature-sensitive polymer under acidic conditions, which needs further verification. In conclusion, the release of drugs from gels is influenced by many factors, including the hydrophilicity of the gel and the drug, the pH of the release environment and the temperature of the release environment. As the hydrophilicity of the gel increases, the swelling increases, and the ratio of the competing drug forces between water and gel increases, the drug is more easily released from the gel [48]. As can be seen in Figure 5 (b), the fluorescence intensity of the membrane increases with the increase of AMB release, so the degree of drug release can be judged by the fluorescence change. This is due to the internal filtering effect of TPE and AMB. With the release of AMB, the excitation light of TPE becomes stronger and stronger, making the emission light of TPE stronger and stronger [9, 49, 50].

In addition, the drug release data in vitro were fitted with a one-level kinetic model to explore the mechanism of drug release. The fitting results showed that the r of all equations was greater than 0.98, which indicates that the first-order kinetic model could describe the drug release process well.

#### 3.2.2 Biocompatibility

**Figure 6:**
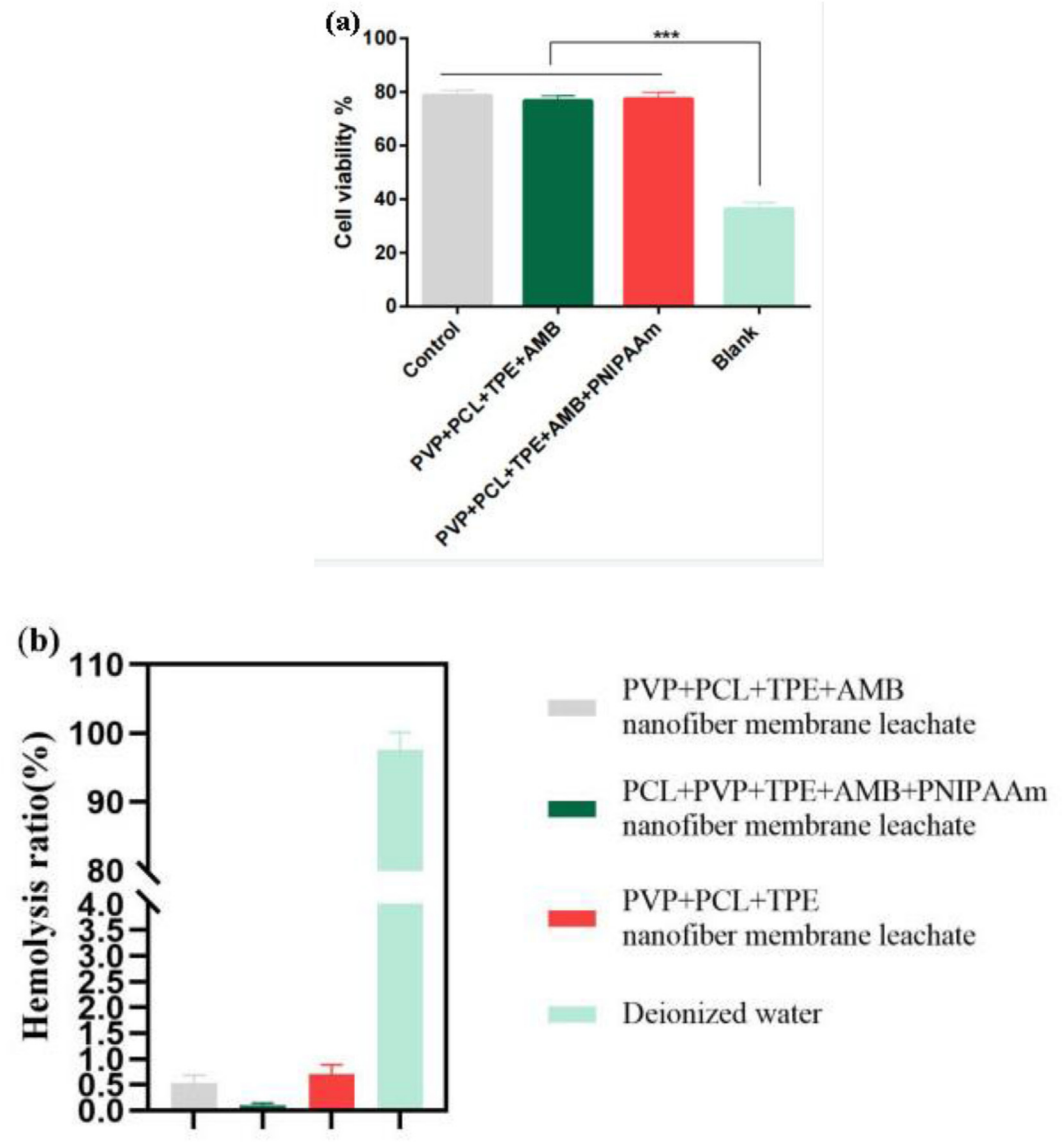
(a) MTT of nanofiber membrane. (b) blood compatibility graph of nanofiber membrane.

Biomedical materials with good biocompatibility are a prerequisite for novel wound dressings [51]. The toxicity of each of the prepared nanofibrous membranes on cells was studied using MTT assay. Figure 6(a) shows the results of the effect of nanofibrous membranes on cell viability. It can be seen that there was essentially no difference in cell survival in the nanofiber membrane group compared to the control group, indicating that the nanofiber membranes were not cytotoxic, which is due to the porous structure of the nanofiber membranes that provides a scaffold for cell growth, thus promoting cell growth and proliferation [52]. Furthermore, according to the experimental results in Figure 6(b), it can be seen that all nanofiber membranes exhibit excellent hemocompatibility in wound healing. All these results confirm the good biocompatibility of nanofibrous membranes.

#### 3.2.3 Antifungal test

**Figure 7:**
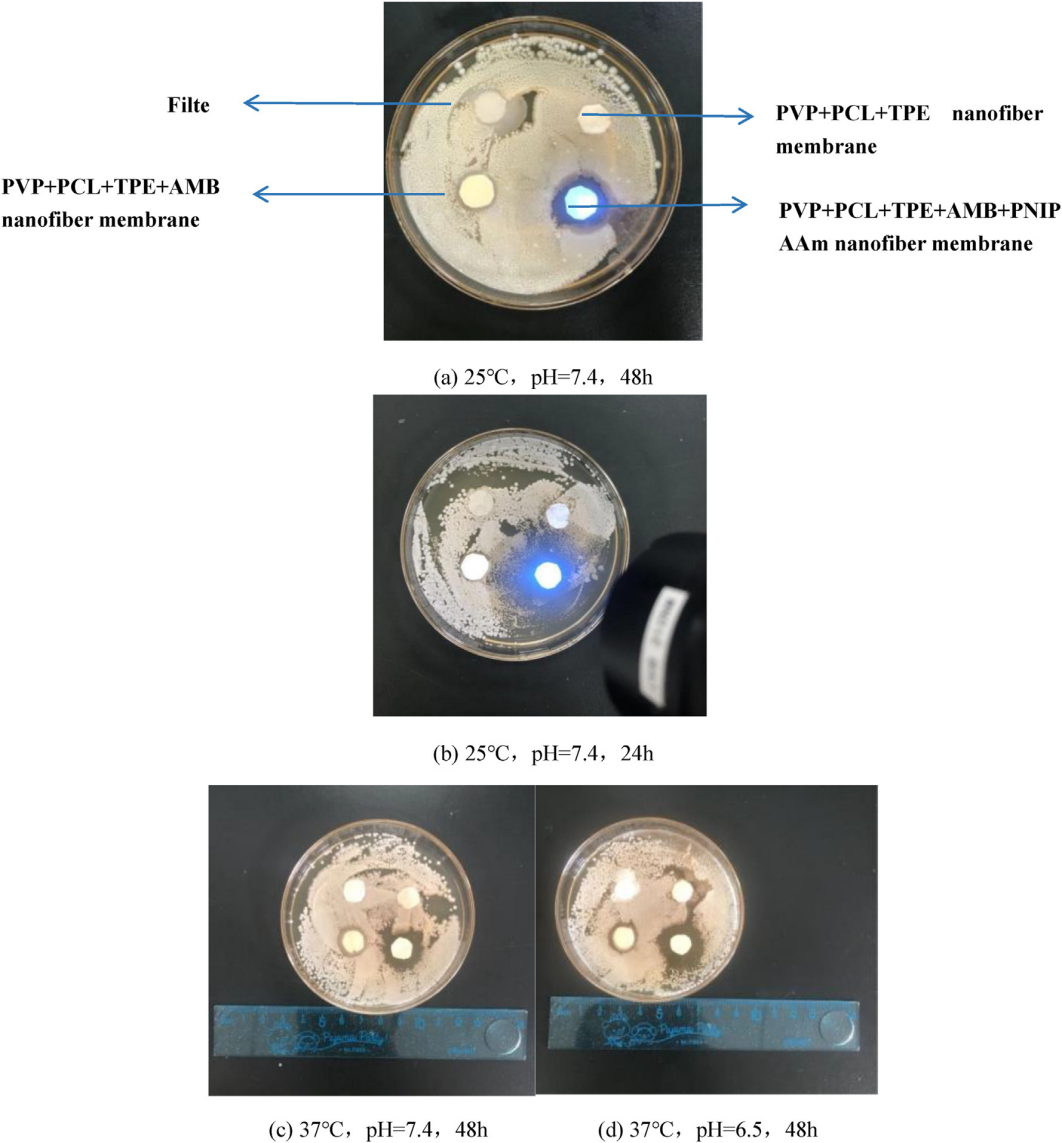
Effect of antifungal properties of nanofiber membrane under different temperature and pH conditions (a) 25 °C, pH = 7.4, 48h. (b) 25 °C, pH = 7.4, 24h. (c) 37 °C, pH = 7.4, 48h. (d) 37 °C, pH = 6.5, 48h.

It is well known that fungal infections may lead to increased exudate at the wound site and slow down the wound healing process [53]. Therefore, the optimal wound dressing material should have inherent antifungal properties to improve wound healing by reducing the number of pathogens and decreasing the inflammatory response of the wound [54, 55]. In this study, surface antifungal activity test was used to evaluate the antifungal activity of all nanofiber films against Candida albicans. The inhibitory effect of plain filter paper sheets and blank nanofiber membranes against Candida albicans can be seen in Figure 7. No inhibition circles appeared in the petri dishes, indicating that they are not antibacterial. Figure 7(a) (c) (d) show the inhibition of Candida albicans in different environments. The figure shows that PCL+PVP+TPE+AMB+PNIPAAm nanofiber membrane and PCL+PVP+TPE+AMB nanofiber membrane group are inhibitory to the fungus, but the inhibition circle of PCL+PVP+TPE+AMB group <PCL+PVP+TPE+ AMB+PNIPAAm, and the inhibition circle width of the effective PCL+PVP+TPE+AMB+PNIPAAm group were 1.62 ± 0.13mm, 2.31 ± 0.09mm, and 2.54 ± 0.18 mm at 25 °C, pH = 7.4, 37 °C, pH = 7.4, 37 °C, and pH = 6.5, respectively, because the nanofiber membranes in the temperature-sensitive polymer-encapsulated drugs were released at different rates under different temperatures and pH conditions to control the drug release for sustained drug release, while the N atoms in the PNIPAAm structure may be positively charged under acidic conditions and combine with the negative charge of the fungal cell wall to enhance the antibacterial properties, which needs to be further verified. Besides, the change of fluorescence intensity of drug-released nanofiber membrane during drug fungal inhibition can be seen in Figure 7 (b).

### 3.3 In vivo assays

#### 3.3.1 Wound healing efficiency

The previous results show that the introduction of PNIPAAm will significantly affect the physical properties of the material. Also, more effective antimicrobial effect was shown in the group containing PNIPAAm, which has great potential in skin wound treatment. Therefore, the wound healing properties of the temperature-sensitive drug-loaded nanofiber membranes were further evaluated in a mouse full-layer wound defect model. Meanwhile, we reflected the drug release based on the measured fluorescence intensity changes of the rat nanofiber membranes to determine the frequency of nanofiber membrane replacement. According to the fluorescence intensity of PVP+PCL+TPE+AMB+PNIPAAm group in Table 5, it can be seen that the fluorescence intensity of nanofiber membrane basically reached the maximum fluorescence intensity at 24h, which means that the drug was basically released completely. Therefore, we can set the time of membrane replacement for the four groups of animals experiments to change once a day.

**Table 5:** Change of fluorescence intensity of nanofiber membrane.

	0h	6h	12h	24h	36h
Change of fluorescence intensity of nanofiber membrane					

**Figure 8:**
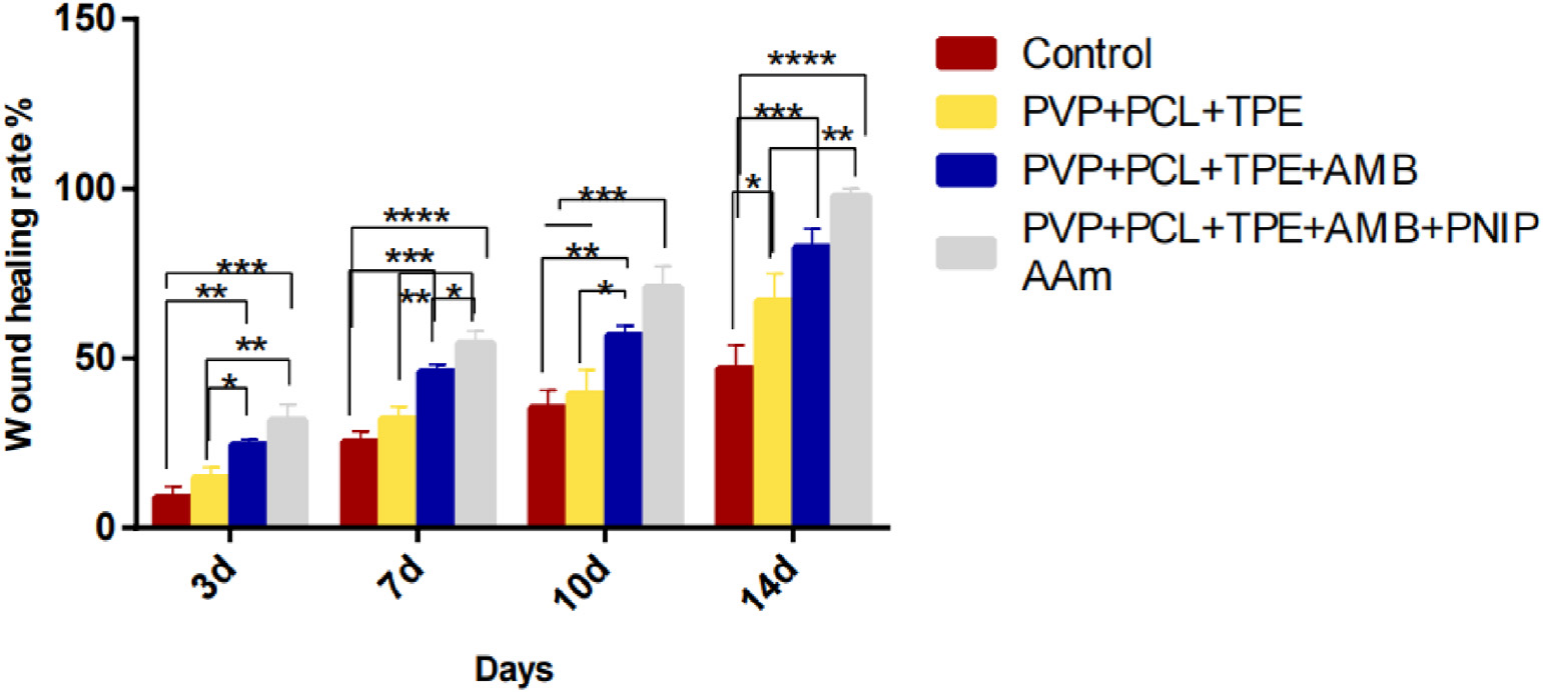
Wound healing rate.

Using the gauze group as the control group, it can be seen in Table 6 and Figure 8 that the nanofiber membrane group showed faster healing performance than the gauze group on days 3, 7, 10 and 14. Specifically, on postoperative day 3, all groups showed different degrees of wound area reduction, and when compared with the control gauze group, the PNIPAAm group showed the largest wound shrinkage area (46.98%, P < 0.05), suggesting that the temperature-sensitive drug-loaded nanofiber membrane had a higher effect on promoting wound healing. On day 7, the difference between the temperature-sensitive drug-loaded nanofiber film group and the control group was more significant (P < 0.05), while the PNIPAAm group also showed a more significant difference than the AMB-only group (P < 0.05), and the wound healing in the drug-added group was also more significant than that in the blank nanofiber film group (P < 0.05), indicating that the therapeutic effect of both drug-loaded groups was better than that of the blank nanofiber film group as well as the control gauze group. On day 10, the difference in wound contraction was more pronounced in the blank nanofiber membrane and control groups compared with the PNIPAAm group (P < 0.05) and the AMB alone group (P < 0.05). On day 14, it was observed that the wounds treated with PVP+PCL+TPE+AMB+PNIPAAm nanofibrous membrane group were completely closed and essentially scar free, and the wounds treated with PVP+PCL+TPE+AMB group were basically healed, while the wounds treated with the control group were not completely healed, with even more obvious scars being formed. The difference in wound healing between the control and PVP+PCL+TPE groups and the two drug-loaded groups was more significant (P < 0.05).

**Table 6:** Wound healing diagram.

	Control	PVP+PCL+TPE	PVP+PCL+TPE+AMB	PVP+PCL+TPE+AMB+PNIPAAm
0d				
3d				
7d				
10d				
14d

These quantitative data of wound area indicate that the PVP+PCL+TPE+AMB+PNIPAAm group had better wound healing than the PVP+PCL+TPE+AMB group, PVP+PCL+TPE group, and gauze group. The trauma dressing based on PVP+PCL+TPE+AMB+PNIPAAm group could accelerate all the healing stages due to the synergistic effect of temperature-sensitive polymer and AMB. The PVP+PCL+TPE+AMB+PNIPAAm group showed better therapeutic efficacy throughout the healing phase compared to the other groups due to the permeability and absorption of water and air by the temperature-sensitive polymer with appropriate porosity and water absorption, maintaining a partially fixed moist environment by absorbing trauma effluent, while the temperature-sensitive polymer also acted as a slow and controlled drug release. The fluorescence intensity of the membrane allows us to keep track of the drug release in real time and replace the nanofiber membrane in time to keep the wound in a state of drug release. Thus, PVP+PCL+TPE+AMB+PNIPAAm nanofiber membranes with suitable physical structure and chemical properties can promote wound healing, and their therapeutic effect is better than the other three groups.

#### 3.3.2 Histological evaluation

Histological analysis was performed to further differentiate the effect of the dressings on wound healing in each group. The main observations were inflammatory cell infiltration, fibroblast migration, connective tissue synthesis and re-epithelialization. As seen in Table 7, a mild acute inflammatory response on day 3, fibroblasts and inflammatory cells in these four groups migrated to the wound site. More importantly, the remaining three groups showed fewer inflammatory cells and more fibroblasts around the damaged area compared to the control group. As the wound healed further, all four groups showed fewer inflammatory cells on day 7, with moderate epidermal thickening of local tissue and repair of granulation tissue proliferation in the P-loaded nanofibrous membrane group, with more capillaries and fibroblasts visible (blue arrows), accompanied by numerous neutrophil and single nucleated cell infiltrates. Compared with the control group, the fibroblasts were well arranged and a small amount of collagen fibers were visible in the interstitium. On day 10, compared with the control group and the blank nanofiber film group, the two drug-loaded nanofiber film groups had new capillary masses, a large number of collagen fibers were visible, inflammatory cell infiltration was reduced, and multiple follicle-like structures were visible. In the temperature-sensitive drug-loaded nanofiber film group, the new epidermis was obviously thickened, the crust basically disappeared, collagen fibers were abundant, follicle-like structures were clearly visible, wound healing was obvious, no bleeding was seen in the tissue, inflammatory cell infiltration could be seen, fibroblasts were few, gelatin material was absorbed obviously, and it could promote the transfer of inflammatory cells into gelatin. On day 14, a large number of fibroblasts were repaired in the control group, collagen fibers were less formed, and wound healing was not obvious. In the blank nanofibrous membrane group, collagen fibers were abundant and dense, fibroblasts were fewer, wound healing was obvious, inflammatory cells were visible, and follicle-like structures were visible. The skin was normal, wound healing was obvious, collagen fibers were abundant and parallel to the wound, collagen fibers were more dense, and vascular density was normal [56, 57, 58].

**Table 7:** Histological analysis of the trauma results.

	Control	PVP+PCL+TPE Nanofiber membrane	PVP+PCL+TPE+AMB Nanofiber membrane	PVP+PCL+TPE+AMB+PNIPAAm Nanofiber membrane
3d				
7d				
10d				
14d				

Note: Inflammatory cell infiltration (yellow arrows); capillary and fibroblast proliferation repair (blue arrows); neoplastic epidermis (green arrows); scab (black arrows); capillary mass (red arrows).

In conclusion, based on the in vivo experimental study, the PVP+PCL+TPE+AMB+PNIPAAm group showed the best wound healing properties among these four groups.

#### 3.3.3 Collagen deposition

**Figure 9:**
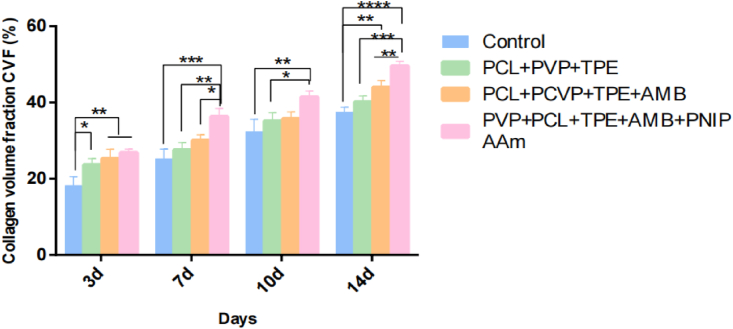
Collagen volume fraction CVF (%).

One of the important markers of wound healing is the total collagen level. Figure 9 shows that on day 3 the collagen volume fraction in the control group was significantly different from the other three nanofibrous membrane groups (P < 0.05), and on days 7 and 10 there were significant differences in the collagen volume fraction in the control, PVP+PCL+TPE, and PVP+PCL+TPE+AMB groups compared to the PVP+PCL+TPE+AMB+PNIPAAm groups (P < 0.05). On day 14, there was a significant difference between the control group and the PVP+PCL+TPE+AMB group (P < 0.05), and the other three groups had more significant differences in collagen volume fraction compared with the PVP+PCL+TPE+AMB+PNIPAAm group (P < 0.05). Throughout the healing process, higher collagen levels were demonstrated compared with the other three groups (P < 0.05), indicating that the PVP+PCL+TPE+AMB+PNIPAAm group had a better healing effect than the other three groups. In conclusion, combined with the wound healing rate, H&E staining results and collagen deposition analysis, the therapeutic effect of PVP+PCL+TPE+AMB+PNIPAAm group was better than the other three groups, indicating that the wound dressing of PVP+PCL+TPE+AMB+PNIPAAm group greatly promoted the wound healing process [22, 59].

#### 3.3.4 Immunohistochemistry

**Figure 10:**
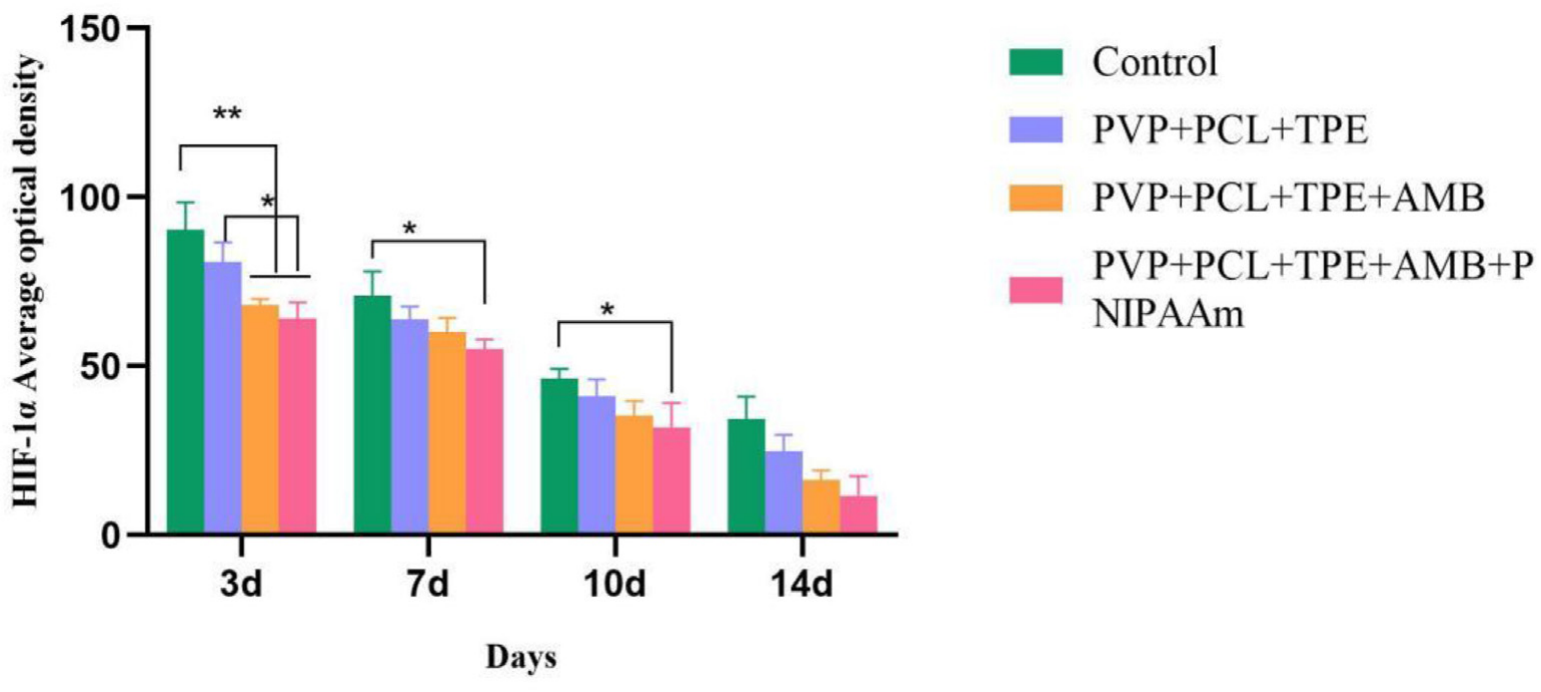
HIF-1α Average optical density (Mean Density).

**Figure 11:**
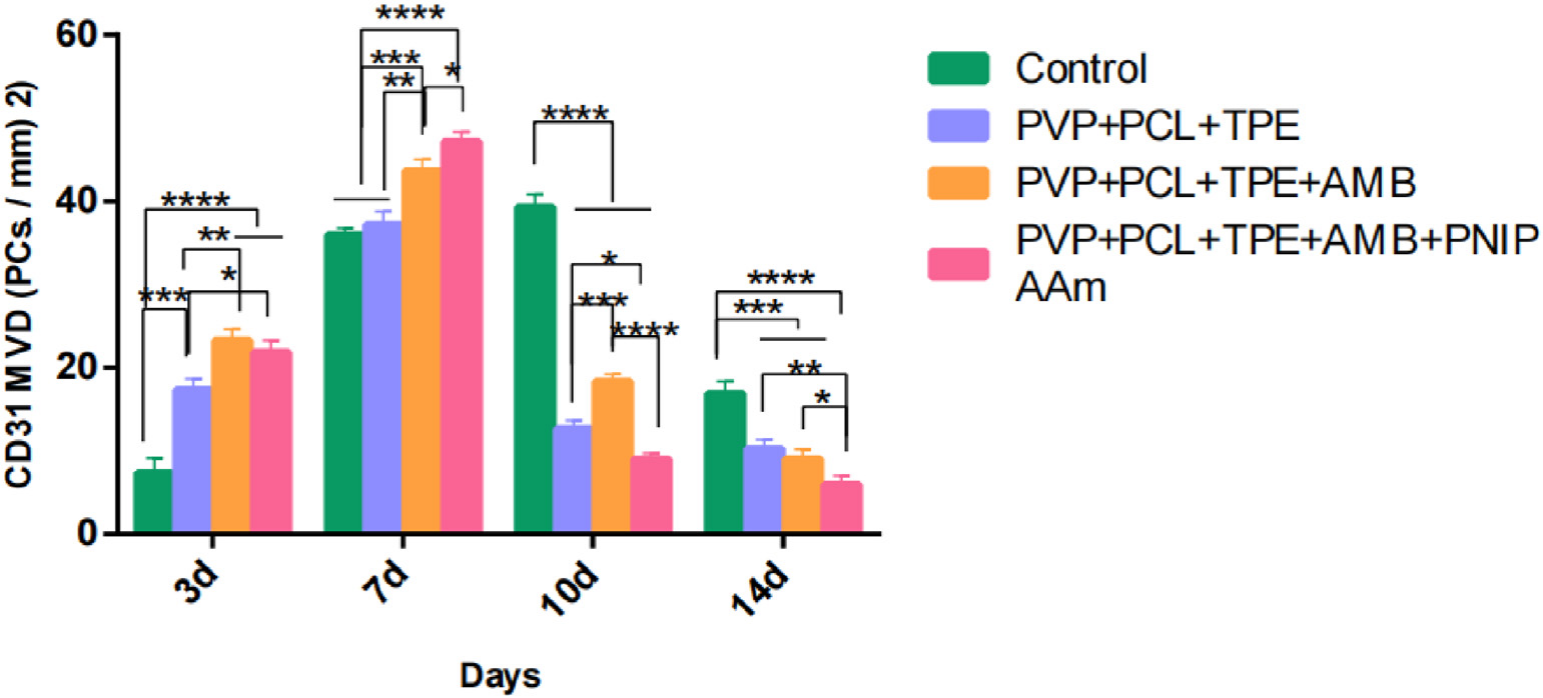
Number of blood vessels per unit area of CD31 MVD (PCs./mm) ^2^).

During wound healing, many essential cellular activities are regulated by cytokines that mainly regulate the initiation and arrest of periwound cells. Therefore, we chose human hypoxia-inducible factor (HIF-1α) as an indicator to verify the therapeutic effect of PVP+PCL+TPE+AMB+PNIPAAm nanofibrous membrane for the prevention of inflammatory infections by immunofluorescence staining. As shown in Figures 10 and 11, on day 3, there was a significant difference between the control group and the PVP+PCL+TPE+AMB group compared with the PVP+PCL+TPE+AMB+PNIPAAm group (P < 0.05). There was also a significant difference between the blank nanofiber membrane group and the PVP+PCL+TPE+AMB+PNIPAAm group (P < 0.05). On days 7 and 10, there was a significant difference between the control group and the PVP+PCL+TPE+AMB+PNIPAAm group (P < 0.05). HIF-1α expression was lowest in the PVP+PCL+TPE+AMB+PNIPAAm group on day 14, which may be due to the fact that the nanofiber membrane, which was changed once a day according to the change in fluorescence intensity, kept the wound in an environment of drug release and reduced inflammation, while the nanofiber membrane had dense voids that isolated bacteria to reduce inflammation [60, 61]. In addition to preventing infection, angiogenesis in the wound is further estimated. Platelet-endothelial cell adhesion molecule (CD31) was selected as an index to evaluate wound repair, and on day 3, CD31 of neovascularization was significantly higher in the PVP+PCL+TPE+AMB group and PVP+PCL+TPE+AMB+PNIPAAm group than in the control and blank nanofiber membrane groups (P < 0.05). On day 7, angiogenesis was significantly lower in the control and blank nanofibrous membrane groups than in the PVP+PCL+TPE+AMB and PVP+PCL+TPE+AMB+PNIPAAm groups (P < 0.05). On days 10 and 14, CD31 of nanofibrous membranes decreased in all three groups, which was significantly different from the control group (P < 0.05). CD31 decreased faster in the PVP+PCL+TPE+AMB+PNIPAAm group compared with the other three groups (P < 0.05). This may be due to the synergistic effect of AMB and PNIPAAm, which played a stronger role in the early stage of wound healing, promoting angiogenesis and accelerating wound healing. In conclusion, the PVP+PCL+TPE+AMB+PNIPAAm group significantly accelerated the wound healing process by simultaneously reducing the production of HIF-1α and upregulating the expression of CD31.

## 4 Conclusion

A series of antifungal nanofibrous membranes were developed by electrospinning temperature-sensitive polymers and antibiotic solutions. Experiments in vivo showed that these multifunctional nanofibrous membrane wound dressings could significantly accelerate the wound healing process. Blood compatibility and cytocompatibility were confirmed by co-culture with red blood cells or NIH/3T3 cells, respectively. In an in vivo wound healing test, the PVP+PCL+TPE+AMB+PNIPAAm group exhibited faster wound healing rates than the other three groups.

In addition, wounds treated with the PVP+PCL+TPE+AMB+PNIPAAm group showed less inflammatory infiltration, higher density of fibroblasts, thicker granulation tissue and thicker collagen deposition. Immunofluorescence staining for HIF-1α and CD31 showed that in wounds treated with the PVP+PCL+TPE+AMB+PNIPAAm group, HIF-1α expression was the lowest, while CD31 expression was the highest. This implies that the PVP+PCL+TPE+AMB+PNIPAAm group promoted the regeneration of blood vessels and reduced the production of periwound pro-inflammatory factors. In conclusion, this multifunctional PVP+PCL+TPE+AMB+PNIPAAm nanofibrous membrane wound dressing is a potential candidate for antifungal and therapeutic skin wound healing.

## Declarations

### Author contribution statement

Xinhui Zhai: Conceived and designed the experiments; Performed the experiments; Analyzed and interpreted the data; Contributed reagents, materials, analysis tools or data; Wrote the paper.

Zongyao Cui, Yali Li, Shuang Hou: Performed the experiments.

Weiyang Shen: Conceived and designed the experiments; Contributed reagents, materials, analysis tools or data; Wrote the paper.

### Funding statement

This research did not receive any specific grant from funding agencies in the public, commercial, or not-for-profit sectors.

### Data availability statement

Data included in article/supp. material/referenced in article.

### Declaration of interests statement

The authors declare no conflict of interest.

### Additional information

No additional information is available for this paper.
